# Early Antibiotic Exposure in Low-resource Settings Is Associated With Increased Weight in the First Two Years of Life

**DOI:** 10.1097/MPG.0000000000001640

**Published:** 2017-08-22

**Authors:** Elizabeth T. Rogawski, James A. Platts-Mills, Jessica C. Seidman, Sushil John, Mustafa Mahfuz, Manjeswori Ulak, Sanjaya Shrestha, Sajid B. Soofi, Pablo Penataro Yori, Estomih Mduma, Erling Svensen, Tahmeed Ahmed, Aldo A.M. Lima, Zulfiqar Bhutta, Margaret Kosek, Dennis Lang, Michael Gottlieb, Anita Zaidi, Gagandeep Kang, Pascal Bessong, Eric R. Houpt, Richard L. Guerrant

**Affiliations:** ∗ Department of Public Health Sciences; † Division of Infectious Diseases and International Health, University of Virginia, Charlottesville, VA; ‡ Fogarty International Center, National Institutes of Health, Bethesda, MD; § Christian Medical College, Vellore, India; || International Centre for Diarrhoeal Disease Research, Dhaka, Bangladesh; ¶ Institute of Medicine, Tribhuvan University; # Walter Reed/Armed Forces Research Institute of Medical Sciences Research Unit, Kathmandu, Nepal; ∗∗ Center for International Health, University of Bergen, Bergen, Norway; †† Aga Khan University, Karachi, Pakistan; ‡‡ Asociación Benéfica PRISMA, Iquitos, Peru; §§ Bloomberg School of Public Health, Johns Hopkins University, Baltimore, MD; |||| Haydom Lutheran Hospital, Haydom, Tanzania; ¶¶ Haukeland University Hospital, Bergen, Norway; ## Clinical Research Unit and Institute of Biomedicine, Federal University of Ceara, Fortaleza, Brazil; ∗∗∗ Foundation for the National Institutes of Health, Bethesda, MD; ††† University of Venda, Thohoyandou, South Africa.

**Keywords:** antibiotics, growth, low-resource settings, pediatric enteric disease

## Abstract

**Objectives:**

The potential growth-promoting effects of antibiotics are not well understood among undernourished children in environments with high pathogen exposure. We aimed to assess whether early antibiotic exposure duration and class were associated with growth to 2 years of age across 8 low-resource sites in the MAL-ED birth cohort study.

**Methods:**

We followed 1954 children twice per week from birth to 2 years to record maternally reported antibiotic exposures and measure anthropometry monthly. We estimated the associations between antibiotic exposure before 6 months of age and weight-for-age and length-for-age (LAZ) *z* scores to 2 years. We assessed the impact of class-specific exposures and duration, and compared these results to effects of antibiotic exposures after 6 months of age.

**Results:**

Antibiotic use before 6 months of age was associated with increased weight from 6 months to 2 years, whereas associations with length were less consistent across sites and antibiotic classes. Compared to unexposed children, 2 or more courses of metronidazole, macrolides, and cephalosporins were associated with adjusted increases in weight-for-age of 0.24 (95% confidence interval (CI): 0.04, 0.43), 0.23 (95% CI: 0.05, 0.42), and 0.19 (95% CI: 0.04, 0.35) from 6 months to 2 years, respectively.

**Conclusions:**

Antibiotic use in low-resource settings was most associated with the ponderal growth of children who had multiple exposures to antibiotics with broad spectrum and anaerobic activity in early infancy. Opportunities for rational and targeted antibiotic therapy in low resource settings may also promote short-term weight gain in children, although longer-term physical growth and metabolic impacts are unknown.

**What Is Known**

- Antibiotics are growth-promoting in livestock, and early life exposure to antibiotics has been recently associated with obesity in several high-income countries.
- The effect of antibiotic use may be different among children in highly contaminated environments in low-income settings in which undernutrition is more common than obesity.

**What Is New**

- Antibiotic-associated growth promotion can also occur among children in low-resource settings who are often undernourished.
- Antibiotic use in the first 6 months of life was associated with increased weight, especially among children with 2 or more exposures to macrolides, metronidazole, and cephalosporins.

After decades of antibiotic use to promote growth of livestock in the agricultural industry, researchers have begun to explore the potential role of antibiotic use in promoting growth in humans as well (1). Evidence of the potential for antibiotics to cause long-lasting perturbations of the gut microbiota (2,3) and the role of the microbiota in metabolism (4–6) has suggested that this exposure could be an important research target as a modifiable factor that may be contributing to the obesity epidemic in high-income countries. Antibiotic exposure early in life, during the maturation process of the gut microbiota and enteric immune system, has been hypothesized to have the largest potential impact on growth (6,7). Epidemiologic studies investigating the role of early life antibiotic exposures in high-income settings of Finland (8), United Kingdom (9,10), United States (11,12), The Netherlands (13), and Denmark (14) have shown associations between early antibiotic exposure and increased weight gain in children from 2 to 10 years of age, although some found mixed or negative results (14,15).

In low-income settings, obesity among young children is less common, and poor growth and undernutrition is often a more pressing public health concern. In these settings, antibiotics are recommended by the WHO as part of the treatment for severe acute malnutrition (16), despite the mixed evidence for the impact of antibiotic treatment on recovery and mortality (17,18). Outside of clinical settings, it is not clear whether antibiotic use promotes growth in children who are not acutely malnourished but remain below the average growth curve and reside in environments with high pathogen exposure. In these settings, antibiotics may affect growth through clearance of infections or through modifications of the microbiota, the hypothesized mechanism in high-income settings (19,20). Unique to low-income settings, there is additional confounding in observational studies by the indications for treatment, because some of the most common illnesses resulting in antibiotic treatment (eg, diarrhea) can have long-term negative impacts on growth. An international cross-sectional study that found a positive association between antibiotic treatment during infancy and BMI among boys did not include these factors and was also limited by self-reported heights and weights (21).

To appropriately account for these factors, we leveraged the high-resolution illness and treatment data from The Etiology, Risk Factors, and Interactions of Enteric Infections and Malnutrition and the Consequences for Child Health and Development Project (MAL-ED), a birth cohort study performed at 8 sites in South America, sub-Saharan Africa, and south Asia. Antibiotic use was highly prevalent in almost all sites (22). We aimed to assess whether early antibiotic use in the first 6 months of life was associated with physical growth from 6 months to 2 years of age and determine the impact of antibiotic class and duration of use.

## METHODS

The MAL-ED study was conducted between November 2009 and February 2014 at 8 sites in Dhaka, Bangladesh, Fortaleza, Brazil, Vellore, India, Bhaktapur, Nepal, Naushahro Feroze, Pakistan, Loreto, Peru, Venda, South Africa, and Haydom, Tanzania. Study design and methods have been previously described (23). Children were enrolled within 17 days of birth if their enrollment weight was ≥1500 g, they were not hospitalized, and they did not have severe or chronic conditions. Follow-up was conducted twice per week at home visits until 2 years of age to document illnesses, breast-feeding practices, and antibiotic use. Caregivers reported all oral or injected antibiotics given to their child. Medication packaging and prescriber documentation were used to confirm antibiotic use and class. Non-diarrheal surveillance stool samples were collected monthly and tested for 40 enteropathogens (24). Weight and length were measured monthly, and weight-for-age (WAZ) and length-for-age (LAZ) *z* scores were calculated using the 2006 WHO child growth standards (25). Length measurements from Pakistan were excluded due to poor data quality. Socioeconomic status was assessed biannually and summarized using the child's average WAMI (Water, Assets, Maternal education, Income) score, which is based on monthly household income, maternal education, wealth measured by 8 assets, and access to improved water and sanitation (26). All sites received ethical approval from their respective governmental, local institutional, and collaborating institutional ethical review boards. Written, informed consent was obtained from the caregiver of each child.

### Data and Definitions

Assessment of antibiotic use practices in the MAL-ED cohort has been previously described (22). A new antibiotic course was defined after 2 antibiotic-free days, assuming antibiotics were not received on the 2% of days with missing surveillance information. Diarrhea was defined as maternal report of 3 or more loose stools in 24 hours or at least 1 loose stool with visible blood (27). Respiratory illness was defined as cough or shortness of breath, and was considered an acute lower respiratory infection if accompanied by fieldworker-determined rapid respiratory rate (27). Fever and vomiting were caregiver reported.

### Analysis

We used multivariable linear regression to estimate the association between antibiotic use in the first 6 months of life and monthly WAZ and LAZ from 6 to 24 months of age. Antibiotic exposure was modeled as a continuous measure of duration in days from 0 to 5 months of age and as a categorical variable by number of courses received to assess the potential for a nonlinear dose-response. We also stratified antibiotic effects by sex and site. Generalized estimating equations with robust variance were used to account for correlation between anthropometric measurements within children across time points. Confounding variables for adjustment included baseline characteristics and indications for treatment, and were selected by causal diagram (28) based on expert opinion and a previous analysis of factors associated with antibiotic use in MAL-ED (22). All analyses were adjusted for site, child sex, enrollment WAZ, WAMI score, crowding (people/room in household), maternal height, maternal education, and characteristics of the child's first 6 months of life: percent days exclusively breast-fed; number of diarrhea episodes; days with fever, vomiting, and respiratory illness; and presence of acute lower respiratory infection, bloody stools, and hospitalization. Length models also included enrollment LAZ.

We further explored the effects of class-specific antibiotic exposure use by modeling class-specific exposure as dichotomous (exposed to a specific class on at least 1 day vs not) and as a categorical variable by number of class-specific courses received, using the models above and additionally adjusting for other antibiotic class exposures to isolate class-specific effects. We also explored effect measure modification by malnutrition (stunted and underweight) at 6 months and pathogen burden from 0 to 5 months (presence of *Campylobacter*, enteroaggregative *Escherichia coli*, and *Giardia*, and average number of bacterial pathogens detected (24)) by including interaction terms between the exposures and these variables and estimating subgroup-specific effects.

To assess the period during which the effects of early antibiotic use (before 6 months of age) were manifested, we estimated these effects on anthropometry at different age periods, from 0 to 5, 6 to 11, 12 to 17, and 17 to 24 months, adjusting for the child's anthropometric *z* scores at the beginning of the age period.

To compare early life exposures with later exposures, we used linear regression to estimate the effects of exposures from 6 to 24 months on cross-sectional WAZ and LAZ at 2 years. A child's measurement closest to 24 months, between 23 and 25 months was considered their anthropometry at 2 years. Adjustment variables included the same baseline characteristics as above, including enrollment WAZ, enrollment LAZ (for length models only), illness burden as characterized above over the whole 2 years of follow-up, and antibiotic use in the first 6 months of life. Using these models with cross-sectional outcomes of WAZ and LAZ at 2 years, we demonstrated that the early life effects were insensitive to further statistical adjustment for illnesses and antibiotic use after 6 months of age.

## RESULTS

Across 8 sites in the MAL-ED cohort, 1954 children were followed until at least 6 months of age and had at least 1 subsequent anthropometric measurement. The majority of these children (n = 1736, 88.3%) remained under surveillance and had anthropometric measurements at 2 years. Baseline characteristics, early antibiotic exposure, and growth outcomes differed across sites (Table 1), with the highest antibiotic use in the South Asian sites (22). Mean enrollment weight and length within 17 days of birth across sites were near 1 standard deviation below the WHO standard. All sites except Brazil showed reductions in average WAZ and LAZ over the 2 years of follow-up with overall means at 2 years of −1.06 and −1.71, respectively.

**FIGURE 1:**
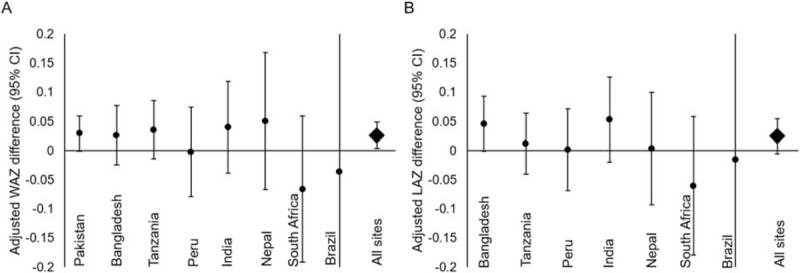
Adjusted weight-for-age (WAZ) (A) and length-for-age (LAZ) (B) *z* score differences associated with a linear 7-day increase in duration of antibiotic exposure in the first 6 months of life among 1954 children followed in the MAL-ED birth cohort until at least 6 months of age with subsequent anthropometry. Sites ordered from greatest to least proportion exposed to antibiotics <6 months. Pakistan: 261/265 (98.5%); Bangladesh: 236/240 (98.3%); Tanzania: 195/245 (79.6%); Peru: 211/269 (78.4%); India: 147/234 (62.8%); Nepal: 129/235 (54.9%); South Africa: 80/259 (13.3%); Brazil: 25/207 (12.1%); All sites: 1284/1954 (65.7%).

A 7-day increase in duration of antibiotic exposure in the first 6 months of life was associated with an adjusted 0.03 (95% confidence interval [CI]: 0.00, 0.05) higher WAZ from 6 months to 2 years of age compared to unexposed children. There was no difference between boys and girls, and this association was largely consistent across sites, except in Brazil and South Africa, where antibiotic use was least common and the estimates are least precise (Fig. 1A). The largest differences in WAZ were associated with children who received >3 courses in the first 6 months (Supplemental Digital Content 2, Fig. 2A). Children who received >3 courses of antibiotics in the first 6 months of life had an average 0.18 (95% CI: 0.06, 0.30) higher WAZ from 6 months to 2 years compared to children receiving 3 courses or less. This effect was largest in Pakistan (WAZ difference: 0.46, 95% CI: 0.25, 0.67) and Tanzania (WAZ difference: 0.21, 95% CI: −0.01, 0.43), but these estimates translate to relatively small differences in weight (600 and 280 g at 2 years, respectively).

The associations of duration of antibiotic exposure with LAZ were more varied across sites (Fig. 1B). There was no difference in LAZ between children exposed to between 0 and 4 courses of antibiotics. Children exposed to 5 or more courses of antibiotics before 6 months had a slightly higher but nonsignificant increase in LAZ compared to unexposed children (Fig., Supplemental Digital Content 2, Fig. 2B).

In the assessment of class-specific exposure, macrolide and metronidazole use on at least 1 day during the first 6 months were associated with an adjusted 0.14 (95% CI: 0.02, 0.25) and 0.17 (95% CI: 0.04, 0.31) increase in WAZ, respectively. Cephalosporins, fluoroquinolones, and penicillins were associated with smaller and nonsignificant increases in WAZ. Associations with LAZ were generally smaller and not statistically significant for any antibiotic class (Table 2). Although the effects of macrolides and cephalosporins were close to null if only 1 course was received, 2 or more courses were associated with an adjusted increase of 0.23 (95% CI: 0.05, 0.42) and 0.19 (95% CI: 0.04, 0.35) in WAZ, respectively (Table 2). Metronidazole was associated with increases in WAZ both when children were exposed to only 1 course (WAZ difference: 0.14, 95% CI: −0.00, 0.29) and 2 or more courses (0.23, 95% CI: 0.05, 0.42). In contrast, a dose-response pattern was absent for the effects on LAZ, except for cephalosporins, in which 2 or more courses of cephalosporins were associated with a 0.19 (95% CI: 0.01, 0.37) increase in LAZ (Table 2). There was no statistical evidence for effect measure modification by malnutrition or pathogen burden in surveillance stools (not shown).

The weight and length increases associated with antibiotic use before 6 months of age occurred during the exposure window and up to 1 year later at 18 months of age. By 6 months, children who were exposed to >3 courses of antibiotics had a 0.09 (95% CI: −0.04, 0.22) greater WAZ compared to children receiving 3 courses or less (Supplemental Digital Content 3, Fig. 3A). These children had further gains of 0.08 (95% CI: −0.06, 0.06) and 0.04 (95% CI: −0.02, 0.05) in WAZ compared to children receiving 3 courses or less from 6 to 11 and 12 to 17 months, respectively. Adjusting for their WAZ at 18 months, there was no further difference in WAZ from 18 to 24 months associated with early antibiotic use (WAZ difference: 0.00, 95% CI: −0.04, 0.05). Children who did not, however, receive early antibiotics did not catch-up during this period such that the majority of the overall WAZ difference was maintained to 24 months. More than 3 courses of antibiotics was associated with a similar increase in LAZ by 6 months (0.08, 95% CI: −0.05, 0.21), but there were no associations in periods after 6 months (Supplemental Digital Content 3, Fig. 3B) or overall as shown above.

In contrast to the antibiotic effects in early infancy, duration of antibiotic exposure after 6 months of age were not associated with cross-sectional WAZ or LAZ at 2 years (Supplemental Digital Content 4, Table), adjusting for exposure before 6 months and illnesses across the first 2 years of life. Among the antibiotic classes, only fluoroquinolones were associated with increases in size at 2 years of age. Two or more courses of fluoroquinolones after 6 months of age were associated with an adjusted increase of 0.21 (95% CI: 0.05, 0.37) in WAZ at 2 years (Supplemental Digital Content 4, Table). The associations with other antibiotic classes were near the null and/or did not show a dose-response trend.

## DISCUSSION

We demonstrate that the growth-promoting phenomenon of early life antibiotic exposure among healthy children is not unique to high-resource settings and can also occur in populations with low average weights and lengths. Antibiotic exposure was associated with increases in weight in the MAL-ED cohort, and this effect was limited to early exposures in the first 6 months of life, which is consistent with previous studies (8,9,13,14). Associations of antibiotics with length were generally smaller and inconsistent across sites and drug classes. The greatest increases in WAZ were associated with >3 courses of exposure and when multiple treatment courses of macrolides, metronidazole, and cephalosporins were received.

A larger impact of macrolides compared to other antibiotics on BMI was similarly documented in a retrospective US study (12). Broad-spectrum antibiotics were associated with early childhood obesity in the United States, whereas narrow-spectrum antibiotics (penicillins) were not (11). Two previous trials of metronidazole treatment in the general pediatric population in Guatemala (1982) (29) and in malnourished children in Jamaica (1993) (30) found an association between metronidazole and improved growth. Although metronidazole is considered narrow-spectrum, its anaerobic activity may be particularly destructive to the gut microbiota. Antibiotics with anaerobic activity have been associated with growth in a dose-dependent manner in the United Kingdom, whereas antibiotics without anaerobic activity were not (10).

Our results provide support for the hypothesized mechanism through the gut microbiota. The influence of antibiotics on the diversity and composition of the gut microbiota can persist long after treatment is completed (2,31), especially among infants (32,33). Altered microbiota can modify metabolism and intestinal inflammation and immunity resulting in increased energy harvest from the diet (4,6,34). Murine models suggest a causal association between the microbiota and obesity; when antibiotic-altered microbiota from overweight mice were transferred to germ-free mice, these mice experienced the same growth and immune response phenotypes as the donor mice (6).

Because the effect of early life antibiotics did not, however, affect growth rates after 18 months of age, the impact of an altered microbiota may be short lived. This evidence that the microbiota is not necessarily permanently “reprogrammed” to cause increased growth rates long after exposure is supported by a recent large study in the United States that found that early antibiotic use was not associated with higher rates of weight gain after the exposure period (15). Their results may be explained by their analytic exclusion of antibiotic-induced weight gain during the exposure period. Taken together, the evidence suggests that short-term boosts in growth due to antibiotics may be more relevant than long-term changes in growth rates.

Still, because the unexposed children in the MAL-ED cohort did not complete catch-up growth, such that the weight difference associated with antibiotic exposure was still present at 24 months, these exposures may have sustained impact on a child's attained size. Short-term growth and attained size have been negatively associated with hospitalization and mortality (35), and positively with cognitive outcomes such as IQ, years of schooling, and income (36), respectively. The first 2 years of life are an especially critical period (37), and even modest improvements in weight gain during this time may be beneficial. Increased relative weight gain in the first 2 years of life, beyond what was expected from linear growth, was, however, not associated with improvements in human capital in Brazil (36), which suggests linear growth may ultimately be more important for long-term cognitive outcomes.

The observed effects limited to exposure in the first 6 months suggest that antibiotic-related modifications of the microbiota, which are most detrimental early in microbiota development, may be more important than clearance of bacterial enteropathogens, which increase in prevalence across the first 2 years of life. If treatment of infections were the main mechanism, we would expect antibiotic exposure after 6 months of age to also have an impact. Furthermore, we did not find that children with more bacterial pathogens before 6 months of age had larger improvements in WAZ associated with antibiotics than those without bacterial pathogens. Pathogen prevalence in the MAL-ED study was, however, high, especially for *Campylobacter* and enteroaggregative *E coli* in the first 6 months of life (38,39), and clearance of enteropathogens may also be contributing to improved growth in these children. Direct analysis of the microbiome in antibiotic exposed and unexposed children would clarify growth-promoting mechanisms.

This analysis improves on previous studies of the relationship between antibiotics and child growth in low-resource settings. The cross-sectional study conducted in 5 nonaffluent countries (Nigeria, India, Indonesia, Thailand, and Syria) relied on caregiver recall of antibiotic exposures at a minimum of 4 years after exposure and caregiver report of anthropometry (21). A previous analysis of an observational birth cohort in India was unable to assess antibiotic class and had partially missing information on antibiotics for nondiarrheal illnesses (40). A systematic review of randomized controlled trials in low- and middle-income countries only assessed antibiotic treatment for trial-specific indications and did not consider cumulative antibiotic exposure in early life (41). In contrast, MAL-ED included prospective follow-up for complete antibiotic information and monthly anthropometry measurement by trained fieldworkers.

This analysis was limited by the inability to assess long-term growth outcomes 5 to 10 years after exposure. This may explain why there was no observable effect of antibiotic exposure after 6 months of age, which may only become apparent later in childhood (9,12,14). This may also explain why the effects on length were smaller since length represents a longer-term growth process. In addition, the precision of estimates was highly dependent on frequency of use, which varied for different antibiotic classes across sites. Because MAL-ED was an observational study, we cannot eliminate the potential for unmeasured confounding, for example, by mode of delivery, which was not recorded. Our analyses, however, accounted for factors associated with antibiotic use previously identified in MAL-ED (22) and each child's detailed illness history. Because we would expect children with more illnesses to be exposed to more antibiotics and have poorer growth, residual confounding by illnesses would likely bias our estimates toward the null, such that our estimates would be conservative.

An association between antibiotics and weight gain in high-income settings is often discussed as a negative side effect of treatment since obesity is a growing public health problem and particularly pernicious among children. The cost-benefit analysis may, however, be different in low-resource settings where children may benefit from improvements in growth and are commonly infected with enteropathogens even in the absence of diarrhea (38). On the contrary, overuse of antibiotics is a major concern worldwide and can lead to adverse events, drug toxicity, and antimicrobial resistance. Antibiotic exposure can also cause antibiotic-associated diarrhea (42), alter intestinal immune function, increase intestinal permeability, and increase risk of systemic infections and subsequent diarrhea (32,43–46). Furthermore, increased weight gain may not be an unmitigated positive if antibiotic-induced changes to the microbiota lead to increased risk for obesity or metabolic syndrome later in life (6). It is unknown whether antibiotic-induced weight gain in these settings is equivalent in terms of developmental impact to similar gains achieved by appropriate nutrition and illness management. Therefore, the total impact of antibiotic exposure early in life among children in low-resource settings is unknown and may be mixed. Reduction of inappropriate antibiotic use must be a public health priority, although opportunities for rational and targeted antibiotic therapy may provide additional benefit by promoting weight gain in these children.

## Supplementary Material

Supplemental Digital Content

## Supplementary Material

Supplemental Digital Content

## Supplementary Material

Supplemental Digital Content

## Supplementary Material

Supplemental Digital Content

## Figures and Tables

**TABLE 1:** Baseline characteristics and antibiotic use by site among 1954 children in the MAL-ED cohort who were followed until at least 6 months of age with subsequent anthropometry

	Dhaka, BangladeshNo. (%)	Fortaleza, BrazilNo. (%)	Vellore, IndiaNo. (%)	Bhaktapur, NepalNo. (%)	Loreto, PeruNo. (%)	Naushahro Feroze, PakistanNo. (%)	Venda, South AfricaNo. (%)	Haydom, TanzaniaNo. (%)	OverallNo. (%)
Children followed until 6 mo with subsequent anthropometry	240	207	234	235	269	265	259	245	1954
Children with anthropometry at 2 years	212 (87.6)	168 (80.4)	227 (96.2)	228 (96.6)	200 (74.1)	249 (94.0)	238 (91.2)	214 (86.3)	1736 (88.3)
Female sex	122 (50.8)	101 (48.8)	128 (54.7)	109 (46.4)	123 (45.7)	136 (51.3)	129 (49.8)	124 (50.6)	972 (49.7)
Crowding in the home (2+ people per room)	225 (93.8)	29 (14.0)	185 (79.0)	93 (39.6)	100 (37.2)	229 (86.4)	35 (13.5)	107 (43.7)	1003 (51.3)
Maternal education < 6 years	152 (63.3)	27 (13.0)	82 (35.3)	60 (25.5)	60 (22.3)	218 (82.3)	6 (2.3)	94 (38.4)	699 (35.8)
Monthly income <150 USD	157 (65.4)	7 (3.4)	216 (92.3)	114 (48.5)	188 (69.9)	145 (54.7)	51 (19.7)	242 (98.8)	1120 (57.3)
Median months of exclusive breast-feeding (IQR)	5.0 (3.8, 5.7)	2.6 (1.3, 4.3)	3.5 (2.5, 4.6)	3.0 (1.5, 4.4)	2.7 (1.0, 4.3)	0.5 (0.3, 0.7)	1.0 (0.6, 1.7)	1.8 (1.1, 2.7)	2.2 (0.9, 4)
Courses of antibiotics before 6 mo									
0	4 (1.7)	182 (87.9)	87 (37.2)	106 (45.1)	58 (21.6)	4 (1.5)	179 (69.1)	50 (20.4)	670 (34.3)
1	18 (7.5)	25 (12.1)	64 (27.4)	90 (38.3)	71 (26.4)	14 (5.3)	55 (21.2)	76 (31.0)	413 (21.1)
2	36 (15.0)	0 (0)	36 (15.4)	26 (11.1)	71 (26.4)	25 (9.4)	21 (8.1)	54 (22.0)	269 (13.8)
3+	182 (75.8)	0 (0)	47 (20.1)	13 (5.5)	69 (25.7)	222 (83.8)	4 (1.5)	65 (26.5)	602 (30.8)
Median days of antibiotics before 6 mo (IQR)	26 (15, 38)	0 (0, 0)	4 (0, 9)	3 (0, 7)	7 (3, 13)	31 (16, 48)	0 (0, 2)	8 (3, 16)	6 (0, 18)
At least 1 day of class-specific antibiotic exposure before 6 mo									
Penicillins	202 (84.2)	18 (8.7)	93 (39.7)	61 (26.0)	173 (64.3)	151 (57.0)	59 (22.8)	150 (61.2)	907 (46.4)
Cephalosporins	158 (65.8)	5 (2.4)	62 (26.5)	27 (11.5)	33 (12.3)	205 (77.4)	1 (0.4)	2 (0.8)	493 (25.2)
Macrolides	143 (59.6)	1 (0.5)	21 (9)	28 (11.9)	86 (32)	79 (29.8)	7 (2.7)	6 (2.5)	371 (19.0)
Metronidazole	8 (3.3)	0 (0)	11 (4.7)	37 (15.7)	2 (0.7)	156 (58.9)	3 (1.2)	59 (24.1)	276 (14.1)
Sulfonamides	3 (1.3)	1 (0.5)	22 (9.4)	19 (8.1)	45 (16.7)	73 (27.6)	10 (3.9)	55 (22.5)	228 (11.7)
Fluoroquinolones	34 (14.2)	0 (0)	16 (6.8)	4 (1.7)	1 (0.4)	9 (3.4)	0 (0)	2 (0.8)	66 (3.4)
*Campylobacter* detection before 6 mo	128 (53.3)	27 (13.0)	99 (42.3)	90 (38.3)	103 (38.3)	175 (66.0)	105 (40.5)	147 (60.0)	874 (44.7)
EAEC detection before 6 mo	166 (69.2)	150 (72.5)	178 (76.1)	147 (62.6)	107 (39.8)	219 (82.6)	161 (62.2)	223 (91.0)	1351 (69.1)
*Giardia* detection before 6 mo	3 (1.3)	6 (2.9)	15 (6.4)	7 (3.0)	19 (7.1)	99 (37.4)	3 (1.2)	20 (8.2)	172 (8.8)
Mean enrollment WAZ^*^	−1.28	−0.16	−1.30	−0.91	−0.62	−1.42	−0.38	−0.13	−0.78
Mean enrollment LAZ^*^	−1.03	−0.78	−1.03	−0.70	−0.96		−0.71	−1.01	−0.89
Mean WAZ at 2 years	−1.61	0.37	−1.65	−0.93	−0.82	−1.65	−0.51	−1.33	−1.06
Mean LAZ at 2 years	−2.03	−0.07	−1.92	−1.35	−1.89		−1.71	−2.66	−1.71

EAEC = enteroaggregative *E coli*; IQR = interquartile range; LAZ = length-for-age *z* score; USD = United States Dollars; WAZ = weight-for-age *z* score.

^*^Within 17 days of birth.

**TABLE 2:** Adjusted weight-for-age and length-for-age *z* score differences associated with class-specific antibiotic use in the first 6 months of life among 1954 children in the MAL-ED birth cohort

Antibiotic class exposure in first 6 months of life	Number exposed (%)(N = 1954^†^)	Adjusted^*^ WAZ difference(95% CI)	Number exposed (%)(N = 1689^†^)	Adjusted^*^ LAZ difference(95% CI)
Metronidazole				
1 Course	170 (8.7)	0.14 (−0.01, 0.29)	109 (6.5)	0.00 (−0.15, 0.16)
2+ Courses	106 (5.4)	0.24 (0.04, 0.43)	11 (0.7)	0.02 (−0.35, 0.40)
Macrolides				
1 Course	258 (13.2)	0.09 (−0.03, 0.21)	206 (12.2)	0.07 (−0.05, 0.18)
2+ Courses	114 (5.8)	0.23 (0.05, 0.42)	87 (5.2)	−0.00 (−0.20, 0.19)
Cephalosporins				
1 Course	244 (12.5)	0.03 (−0.09, 0.16)	182 (10.8)	0.02 (−0.11, 0.14)
2+ Courses	249 (12.7)	0.19 (0.04, 0.35)	106 (6.3)	0.19 (0.01, 0.37)
Penicillins				
1 Course	450 (23.0)	0.09 (−0.02, 0.19)	382 (22.6)	−0.04 (−0.13, 0.06)
2+ Courses	457 (23.4)	0.07 (−0.04, 0.18)	373 (22.1)	−0.00 (−0.11, 0.10)
Fluoroquinolones (any)	66 (3.4)	0.08 (−0.14, 0.30)	57 (3.4)	0.05 (−0.16, 0.27)
Sulfonamides (any)	228 (11.7)	0.03 (−0.09, 0.15)	155 (9.2)	−0.02 (−0.14, 0.11)

LAZ = length-for-age *z* score; WAZ = weight-for-age *z* score.

^*^Adjusted for other antibiotic classes included in the table, site, child sex, enrollment WAZ, Water, Assets, Maternal education, Income (WAMI) score, crowding (people/room in household), maternal height, maternal education, and characteristics of the child's first 6 months of life: percent days exclusively breast-fed; number of diarrhea episodes; days with fever, vomiting, and respiratory illness; and presence of acute lower respiratory infection (ALRI), bloody stools, and hospitalization. LAZ difference is also adjusted for enrollment LAZ.

^†^Children who were followed in the MAL-ED birth cohort until at least 6 months of age with subsequent anthropometry. LAZ difference estimates exclude Pakistan.
