# Supplementary material for: Early Antibiotic Exposure in Low-resource Settings Is Associated With Increased Weight in the First Two Years of Life
Source: J Pediatr Gastroenterol Nutr. 2017 Aug 22;65(3):350–6. doi: 10.1097/MPG.0000000000001640 (PMC5559187; doi:10.1097/MPG.0000000000001640)
Supplement: Supplemental Digital Content [file jpga-65-350-s001.docx]

**Text, Supplemental Digital Content 1**

**MAL-ED Investigators and Institutional Affiliations**

Angel Mendez Acosta^1^, Rosa Rios de Burga^1^, Cesar Banda Chavez^1^, Julian Torres Flores^1^, Maribel Paredes Olotegui^1^, Silvia Rengifo Pinedo^1^, Mery Siguas Salas^1^, Dixner Rengifo Trigoso^1^, Angel Orbe Vasquez^1^, Imran Ahmed^2^, Didar Alam^2^, Asad Ali^2^, Zulfiqar A Bhutta^2^, Shahida Qureshi^2^, Muneera Rasheed^2^, Sajid Soofi^2^, Ali Turab^2^, Anita KM Zaidi^2,^ Ladaporn Bodhidatta^3^, Carl J Mason^3^, Sudhir Babji^4^, Anuradha Bose^4^, Ajila T George^4^, Dinesh Hariraju^4^, M. Steffi Jennifer^4^, Sushil John^4^, Shiny Kaki^4^, Gagandeep Kang^4^, Priyadarshani Karunakaran^4^, Beena Koshy^4^, Robin P Lazarus^4^, Jayaprakash Muliyil^4^, Mohan Venkata Raghava^4^, Sophy Raju^4^, Anup Ramachandran^4^, Rakhi Ramadas^4^, Karthikeyan Ramanujam^4^, Anuradha Rose^4^, Reeba Roshan^4^, Srujan L Sharma^4^, Shanmuga Sundaram E^4^, Rahul J Thomas^4^, William K Pan^5,6^, Ramya Ambikapathi^6^, J Daniel Carreon^6^, Vivek Charu^6^, Viyada Doan^6^, Jhanelle Graham^6^, Christel Hoest^6^, Stacey Knobler^6^, Dennis R Lang^6, 7^, Benjamin JJ McCormick^6^, Monica McGrath^6^, Mark A Miller^6^, Archana Mohale^6,^ Gaurvika Nayyar^6^, Stephanie Psaki^6^, Zeba Rasmussen^6^, Stephanie A Richard^6^, Jessica C Seidman^6^, Vivian Wang^6^, Rebecca Blank^7^, Michael Gottlieb^7^, Karen H Tountas^7^, Caroline Amour^8^, Eliwaza Bayyo^8^, Estomih R Mduma^8^, Regisiana Mvungi^8^, Rosemary Nshama^8^, John Pascal^8^, Buliga Mujaga Swema^8^, Ladislaus Yarrot^8^, Tahmeed Ahmed^9^, AM Shamsir Ahmed^9^, Rashidul Haque^9^, Iqbal Hossain^9^, Munirul Islam^9^, Mustafa Mahfuz^9^, Dinesh Mondal^9^, Fahmida Tofail^9^, Ram Krishna Chandyo^10^, Prakash Sunder Shrestha^10^, Rita Shrestha^10^, Manjeswori Ulak^10^, Aubrey Bauck^11^, Robert Black^11^, Laura E Caulfield^11^, William Checkley^11,6^, Margaret N Kosek^11^, Gwenyth Lee^11^, Kerry Schulze^11^, Pablo Peñataro Yori^11^, Laura E. Murray-Kolb^12^, A Catharine Ross^12^, Barbara Schaefer^12,6^, Suzanne Simons^12^, Laura Pendergast^13^, Cláudia B Abreu^14^, Hilda Costa^14^, Alessandra Di Moura^14^, José Quirino Filho^14,6^, Alexandre Havt^14^, Álvaro M Leite^14^, Aldo AM Lima^14^, Noélia L Lima^14^, Ila F Lima^14^, Bruna LL Maciel^14^, Pedro HQS Medeiros^14^, Milena Moraes^14^, Francisco S Mota^14^, Reinaldo B Oriá^14,^ Josiane Quetz^14^, Alberto M Soares^14^,Rosa MS Mota^14^, Crystal L Patil^16^, Pascal Bessong^17^, Cloupas Mahopo^17^, Angelina Maphula^17^, Emanuel Nyathi^17^, Amidou Samie^17^, Leah Barrett^18^, Rebecca Dillingham^18^, Jean Gratz^18^, Richard L Guerrant^18^, Eric Houpt^18^, William A Petri, Jr^18^, James Platts-Mills^18^, Rebecca Scharf^18^, Elizabeth T. Rogawski^18^, Binob Shrestha^19^, Sanjaya Kumar Shrestha^19^, Tor Strand^19,15^, Erling Svensen^20,8^

^1^A.B. PRISMA, Iquitos, Peru, ^2^Aga Khan University, Karachi, Pakistan, ^3^Armed Forces Research Institute of Medical Sciences, Bangkok, Thailand, ^4^Christian Medical College, Vellore, India, ^5^Duke University, Durham, NC, USA, ^6^Fogarty International Center/National Institutes of Health, Bethesda, MD, USA, ^7^Foundation for the NIH, Bethesda, MD, USA, ^8^Haydom Lutheran Hospital, Haydom, Tanzania, ^9^icddr,b, Dhaka, Bangladesh, ^10^Institute of Medicine, Tribhuvan University, Kathmandu, Nepal, ^11^Johns Hopkins University, Baltimore, MD, USA, ^12^The Pennsylvania State University, University Park, PA, USA, ^13^Temple University, Philadelphia, PA, USA, ^14^Universidade Federal do Ceara, Fortaleza, Brazil, ^15^University of Bergen, Norway, ^16^University of Illinois at Chicago, IL, USA, ^17^University of Venda, Thohoyandou, South Africa, ^18^University of Virginia, Charlottesville, VA, USA, ^19^Walter Reed/AFRIMS Research Unit, Kathmandu, Nepal, ^20^Haukeland University Hospital, Bergen, Norway
