# Supplementary material for: Early Antibiotic Exposure in Low-resource Settings Is Associated With Increased Weight in the First Two Years of Life
Source: J Pediatr Gastroenterol Nutr. 2017 Aug 22;65(3):350–6. doi: 10.1097/MPG.0000000000001640 (PMC5559187; doi:10.1097/MPG.0000000000001640)
Supplement: Supplemental Digital Content [file jpga-65-350-s002.docx]

**
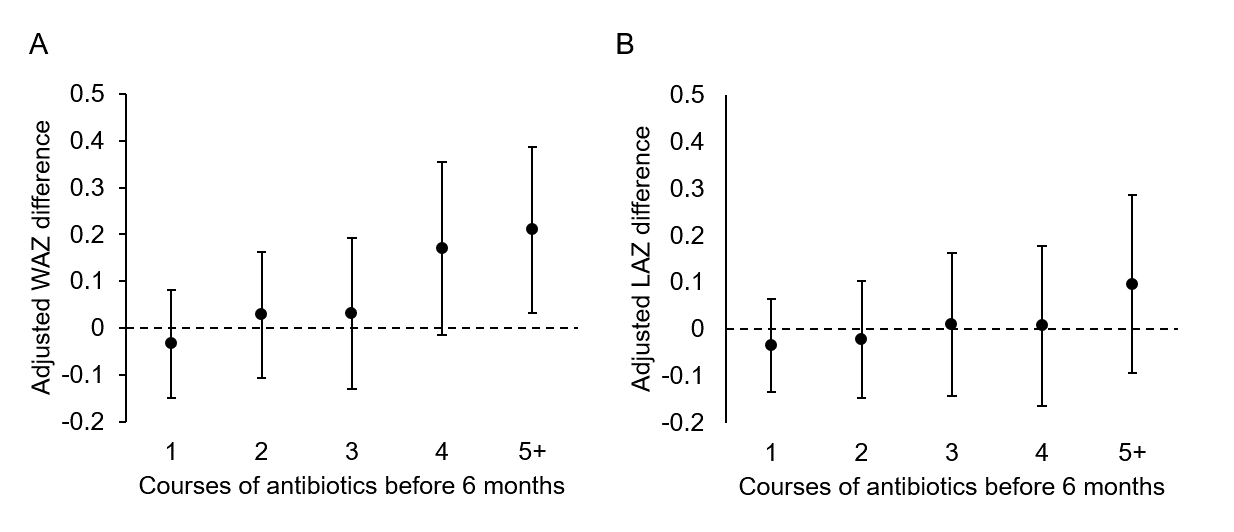
**

**Figure, Supplemental Digital Content 2**. Adjusted difference in anthropometric z-scores associated with number of antibiotic courses received in the first 6 months of life compared to no antibiotic exposure among 1954 children followed in the MAL-ED birth cohort until at least 6 months of age with subsequent anthropometry. A: weight-for-age z-scores (WAZ); B: length-for-age z-scores (LAZ).
