# Supplementary material for: Early Antibiotic Exposure in Low-resource Settings Is Associated With Increased Weight in the First Two Years of Life
Source: J Pediatr Gastroenterol Nutr. 2017 Aug 22;65(3):350–6. doi: 10.1097/MPG.0000000000001640 (PMC5559187; doi:10.1097/MPG.0000000000001640)
Supplement: Supplemental Digital Content [file jpga-65-350-s003.docx]

A B

**Figure, Supplemental Digital Content 3.** Adjusted difference in weight-for-age z-scores (WAZ; A) and length-for-age z-scores (LAZ; B) during 6-month age periods from birth to two years of age associated with antibiotic exposure in the first 6 months of life among 1954 children followed in the MAL-ED birth cohort until at least 6 months of age with subsequent anthropometry. For each estimate, the exposure is receiving more than 3 courses of antibiotics in the first 6 months of life compared to children receiving 3 courses or less. Estimates are adjusted for the attained z-score at the beginning of the age period to isolate the early antibiotic exposure effects during that period.
