# Supplementary material for: Early Antibiotic Exposure in Low-resource Settings Is Associated With Increased Weight in the First Two Years of Life
Source: J Pediatr Gastroenterol Nutr. 2017 Aug 22;65(3):350–6. doi: 10.1097/MPG.0000000000001640 (PMC5559187; doi:10.1097/MPG.0000000000001640)
Supplement: Supplemental Digital Content [file jpga-65-350-s004.docx]

**Table, Supplemental Digital Content 4**. Effects of antibiotic exposure from 6 months to two years of life on cross-sectional WAZ and LAZ at two years of age among 1736 children in the MAL-ED cohort.

|  | Number exposed (%)  (N=1736^†^) | Adjusted* WAZ difference at 2 years  (95% CI) | Number exposed (%)  (N=1487^†^) | Adjusted* LAZ difference at 2 years  (95% CI) |
| --- | --- | --- | --- | --- |
| Duration of exposure in age periods from 6-23 mo. (per 7 day increase) |  |  |  |  |
| 6-11 mo. | 1293 (74.5) | -0.01 (-0.03, 0.02) | 1048 (70.5) | 0.00 (-0.02, 0.03) |
| 12-17 mo. | 1288 (74.2) | 0.00 (-0.03, 0.03) | 1043 (70.1) | -0.00 (-0.03, 0.02) |
| 18-23 mo. | 1210 (69.7) | -0.00 (-0.03, 0.03) | 973 (65.4) | 0.02 (-0.02, 0.05) |
| Antibiotic class from 6-23 mo. |  |  |  |  |
| Macrolides |  |  |  |  |
| 1 course | 273 (15.7) | 0.06 (-0.07, 0.18) | 216 (14.5) | -0.01 (-0.15, 0.13) |
| 2+ courses | 424 (24.4) | 0.01 (-0.14, 0.16) | 389 (26.2) | 0.09 (-0.06, 0.24) |
| Metronidazole |  |  |  |  |
| 1 course | 313 (18.0) | -0.09 (-0.20, 0.02) | 272 (18.3) | -0.09 (-0.21, 0.03) |
| 2+ courses | 357 (20.6) | 0.00 (-0.14, 0.15) | 164 (11.0) | -0.09 (-0.23, 0.06) |
| Cephalosporins |  |  |  |  |
| 1 course | 236 (13.6) | -0.06 (-0.20, 0.07) | 207 (13.9) | 0.01 (-0.13, 0.15) |
| 2+ courses | 514 (29.6) | -0.01 (-0.16, 0.13) | 311 (20.9) | 0.12 (-0.03, 0.27) |
| Fluoroquinolones |  |  |  |  |
| 1 course | 220 (12.7) | 0.15 (0.02, 0.29) | 169 (11.4) | 0.04 (-0.10, 0.19) |
| 2+ courses | 141 (8.1) | 0.21 (0.05, 0.37) | 117 (7.9) | 0.10 (-0.06, 0.25) |
| Penicillins |  |  |  |  |
| 1 course | 340 (19.6) | 0.04 (-0.09, 0.17) | 300 (20.2) | 0.06 (-0.07, 0.20) |
| 2+ courses | 1017 (58.6) | 0.05 (-0.06, 0.19) | 842 (56.6) | -0.01 (-0.14, 0.12) |
| Sulfonamides |  |  |  |  |
| 1 course | 314 (18.1) | 0.01 (-0.10, 0.13) | 248 (16.7) | 0.08 (-0.04, 0.20) |
| 2+ courses | 287 (16.5) | -0.04 (-0.17, 0.08) | 185 (12.4) | 0.04 (-0.10, 0.18) |

*Adjusted for other antibiotic classes included in the table, site, child sex, enrollment WAZ, WAMI score, crowding (people/room in household), percent days exclusively breastfed in the first 6 months of life, maternal height, maternal education, and illness burden during the first two years of life: number of diarrhea episodes, days with fever, vomiting, and respiratory illness, and presence of ALRI, bloody stools, and hospitalization. LAZ difference is also adjusted for enrollment LAZ.

†Children who had anthropometric measurements at two years of age. LAZ difference estimates exclude Pakistan.

WAZ, weight-for-age z-score. LAZ, length-for-age z-score
